# 2-(3,4,5-Trimethoxy­phen­yl)-1*H*-benzimidazole

**DOI:** 10.1107/S1600536808014189

**Published:** 2008-05-17

**Authors:** Aliakbar Dehno Khalaji, Fangfang Jian, Hailian Xiao, William T. A. Harrison

**Affiliations:** a Department of Science, Gorgan University of Agricultrual Sciences and Natural Resources, Gorgan 49189-43464, Iran; b New Materials and Function Coordination Chemistry Laboratory, Qingdao University of Science and Technology, Qingdao 266042, People’s Republic of China; c Department of Chemistry, University of Aberdeen, Meston Walk, Aberdeen AB24 3UE, Scotland

## Abstract

In the title compound, C_16_H_16_N_2_O_3_, the dihedral angle between the mean planes of the aromatic ring systems is 30.90 (15)°. In the crystal structure, the mol­ecules form [010] chains by way of N—H⋯N hydrogen bonds.

## Related literature

For a related structure, see: Rashid *et al.* (2007 ▶). For background, see: Gupta *et al.* (2004 ▶). For reference structural data, see: Allen *et al.* (1987 ▶).
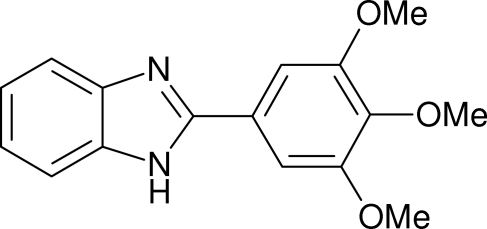


## Experimental

### 

#### Crystal data

C_16_H_16_N_2_O_3_
*M* *_r_* = 284.31
Orthorhombic, 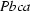

*a* = 8.2270 (16) Å
*b* = 9.5750 (19) Å
*c* = 37.375 (7) Å
*V* = 2944.2 (10) Å^3^
*Z* = 8
Mo *K*α radiation
μ = 0.09 mm^−1^
*T* = 295 (2) K
0.25 × 0.20 × 0.18 mm

#### Data collection

Enraf-Nonius CAD-4 diffractometer
Absorption correction: none
5421 measured reflections
2733 independent reflections
960 reflections with *I* > 2σ(*I*)
*R*_int_ = 0.085
3 standard reflections every 100 reflections intensity decay: none

#### Refinement

*R*[*F* ^2^ > 2σ(*F* ^2^)] = 0.051
*wR*(*F* ^2^) = 0.143
*S* = 0.94
2733 reflections
190 parameters
H-atom parameters constrained
Δρ_max_ = 0.21 e Å^−3^
Δρ_min_ = −0.20 e Å^−3^

### 

Data collection: *CAD-4 Software* (Enraf–Nonius, 1989 ▶); cell refinement: *CAD-4 Software*; data reduction: *NRCVAX* (Gabe *et al.*, 1989 ▶); program(s) used to solve structure: *SHELXS97* (Sheldrick, 2008 ▶); program(s) used to refine structure: *SHELXL97* (Sheldrick, 2008 ▶); molecular graphics: *ORTEP-3* (Farrugia, 1997 ▶); software used to prepare material for publication: *SHELXL97*.

## Supplementary Material

Crystal structure: contains datablocks I, global. DOI: 10.1107/S1600536808014189/bt2706sup1.cif

Structure factors: contains datablocks I. DOI: 10.1107/S1600536808014189/bt2706Isup2.hkl

Additional supplementary materials:  crystallographic information; 3D view; checkCIF report

## Figures and Tables

**Table 1:** Hydrogen-bond geometry (Å, °)

*D*—H⋯*A*	*D*—H	H⋯*A*	*D*⋯*A*	*D*—H⋯*A*
N1—H1*A*⋯N2^i^	0.86	2.07	2.918 (4)	169

Symmetry code: (i) 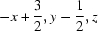.

**Fig. 1.**
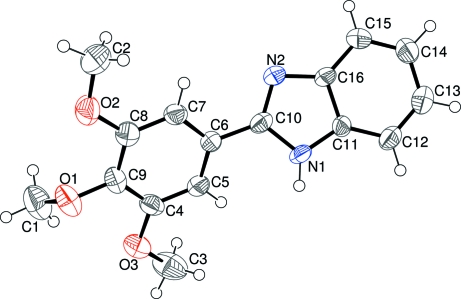
View of the molecular structure of (I) showing 50% displacement ellipsoids (arbitrary spheres for the H atoms).

**Fig. 2.**
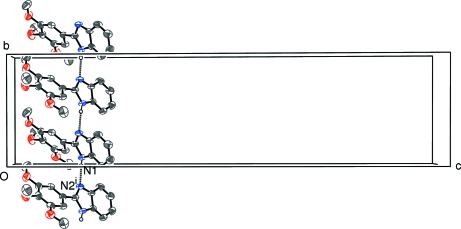
Fragment of a [010] hydrogen bonded chain of molecules in the crystal of (I). Symmetry code: (i) 3/2 - x, y - 1/2, z.

## supplementary crystallographic information

### Comment

The title compound, (I), (Fig. 1), complements substituted imidazoles with biological properties (Gupta *et al.*, 2004). The dihedral angle between the N1/N2/C10—C16 and C4—C9 aromatic ring planes in (I) is 30.90 (15)°. This twisting may help to relieve steric strain between H1a and H5a (H1a···H5a = 2.32 Å) and a number of related 2-phenyl-1*H*-benzimidazoles show a similar dihedral angle between the adjacent ring planes (Rashid *et al.*, 2007). Atoms C1, C2 and C3 in (I) are displaced from the mean plane of the C4—C9 ring by 1.010 (5) Å, 0.115 (5)Å and 0.257 (4) Å, respectively. Otherwise, the geometry of (I) may be regarded as normal (Allen *et al.*, 1987).

In the crystal of (I), an N—H···N hydrogen bond (Table 1) links the molecules into chains propagating in [010] (Fig. 2). There are no aromatic π-π stacking interactions in (I) as the closest centroid-centroid separation of aromatic rings is greater than 5.11 Å, which contrasts with the situation in 2-(4-fluorophenyl)-1*H*-benzimidazole (Rashid *et al.*, 2007) in which both N—H···N and π-π stacking help to establish the packing.

### Experimental

1,2-Phenylenediamine (2 mmol, 216 mg) and 3,4,5-trimethoxybenzaldehyde (2 mmol, 392 mg) were dissolved in methanol (25 ml) at 323 K. The mixture was stirred for 30 min to give a colourless solution. After the solution had been allowed to stand in air for 3 d, colourless blocks of (I) formed, in about 74% yield, on slow evaporation of the solvent at room temperature.

### Refinement

The H atoms were geometrically placed (C—H = 0.93–0.96 Å, N—H = 0.86 Å) and refined as riding with *U*_iso_(H) = 1.2*U*_eq_(carrier) or 1.5*U*_eq_(methyl C).

### Crystal data

**Table:**

C_16_H_16_N_2_O_3_	*F*_000_ = 1200
*M**_r_* = 284.31	*D*_x_ = 1.283 Mg m^−^^3^
Orthorhombic, *P**b**c**a*	Mo *K*α radiation λ = 0.71073 Å
Hall symbol: -P 2ac 2ab	Cell parameters from 25 reflections
*a* = 8.2270 (16) Å	θ = 4–14º
*b* = 9.5750 (19) Å	µ = 0.09 mm^−^^1^
*c* = 37.375 (7) Å	*T* = 295 (2) K
*V* = 2944.2 (10) Å^3^	Block, colourless
*Z* = 8	0.25 × 0.20 × 0.18 mm

### Data collection

**Table:**

Enraf-Nonius CAD-4 diffractometer	*R*_int_ = 0.085
Radiation source: fine-focus sealed tube	θ_max_ = 25.5º
Monochromator: graphite	θ_min_ = 1.1º
*T* = 295(2) K	*h* = −9→0
ω scans	*k* = −11→0
Absorption correction: none	*l* = −44→44
5421 measured reflections	3 standard reflections
2733 independent reflections	every 100 reflections
960 reflections with *I* > 2σ(*I*)	intensity decay: none

### Refinement

**Table:**

Refinement on *F*^2^	Secondary atom site location: difference Fourier map
Least-squares matrix: full	Hydrogen site location: inferred from neighbouring sites
*R*[*F*^2^ > 2σ(*F*^2^)] = 0.051	H-atom parameters constrained
*wR*(*F*^2^) = 0.143	*w* = 1/[σ^2^(*F*_o_^2^) + (0.0483*P*)^2^] where *P* = (*F*_o_^2^ + 2*F*_c_^2^)/3
*S* = 0.94	(Δ/σ)_max_ < 0.001
2733 reflections	Δρ_max_ = 0.21 e Å^−^^3^
190 parameters	Δρ_min_ = −0.20 e Å^−^^3^
Primary atom site location: structure-invariant direct methods	Extinction correction: none

### Special details

**Table:**

Geometry. All e.s.d.'s (except the e.s.d. in the dihedral angle between two l.s. planes) are estimated using the full covariance matrix. The cell e.s.d.'s are taken into account individually in the estimation of e.s.d.'s in distances, angles and torsion angles; correlations between e.s.d.'s in cell parameters are only used when they are defined by crystal symmetry. An approximate (isotropic) treatment of cell e.s.d.'s is used for estimating e.s.d.'s involving l.s. planes.
Refinement. Refinement of *F*^2^ against ALL reflections. The weighted *R*-factor *wR* and goodness of fit *S* are based on *F*^2^, conventional *R*-factors *R* are based on *F*, with *F* set to zero for negative *F*^2^. The threshold expression of *F*^2^ > σ(*F*^2^) is used only for calculating *R*-factors(gt) *etc*. and is not relevant to the choice of reflections for refinement. *R*-factors based on *F*^2^ are statistically about twice as large as those based on *F*, and *R*- factors based on ALL data will be even larger.

### Fractional atomic coordinates and isotropic or equivalent isotropic displacement parameters (Å^2^)

**Table:**

	*x*	*y*	*z*	*U*_iso_*/*U*_eq_	
O1	1.2012 (3)	0.2286 (3)	0.03723 (7)	0.0646 (9)	
O2	0.9428 (3)	0.3940 (3)	0.03507 (7)	0.0591 (9)	
O3	1.2638 (3)	0.0641 (3)	0.09566 (8)	0.0609 (8)	
N1	0.7160 (3)	0.0683 (3)	0.16552 (8)	0.0387 (8)	
H1A	0.7705	−0.0074	0.1623	0.046*	
N2	0.6374 (3)	0.2921 (3)	0.15868 (8)	0.0406 (8)	
C1	1.3578 (5)	0.2888 (6)	0.04310 (12)	0.0800 (15)	
H1B	1.4197	0.2852	0.0213	0.120*	
H1C	1.4134	0.2375	0.0615	0.120*	
H1D	1.3455	0.3843	0.0505	0.120*	
C2	0.8079 (6)	0.4863 (5)	0.03285 (12)	0.0772 (15)	
H2B	0.8153	0.5402	0.0112	0.116*	
H2C	0.8083	0.5479	0.0531	0.116*	
H2D	0.7089	0.4332	0.0327	0.116*	
C3	1.3004 (5)	−0.0119 (5)	0.12749 (11)	0.0772 (15)	
H3A	1.4032	−0.0581	0.1248	0.116*	
H3B	1.2171	−0.0801	0.1317	0.116*	
H3C	1.3055	0.0513	0.1474	0.116*	
C4	1.1200 (5)	0.1366 (4)	0.09509 (12)	0.0446 (11)	
C5	1.0041 (5)	0.1274 (4)	0.12166 (11)	0.0429 (10)	
H5A	1.0203	0.0672	0.1409	0.052*	
C6	0.8635 (4)	0.2074 (4)	0.11992 (10)	0.0368 (9)	
C7	0.8382 (4)	0.2994 (4)	0.09110 (10)	0.0397 (10)	
H7A	0.7448	0.3540	0.0901	0.048*	
C8	0.9539 (5)	0.3074 (4)	0.06435 (11)	0.0422 (10)	
C9	1.0951 (4)	0.2256 (4)	0.06576 (10)	0.0446 (11)	
C10	0.7391 (5)	0.1932 (4)	0.14767 (9)	0.0374 (9)	
C11	0.5910 (4)	0.0866 (4)	0.18929 (10)	0.0338 (9)	
C12	0.5161 (5)	−0.0025 (4)	0.21332 (10)	0.0453 (11)	
H12A	0.5493	−0.0948	0.2158	0.054*	
C13	0.3906 (5)	0.0513 (4)	0.23340 (11)	0.0532 (11)	
H13A	0.3387	−0.0055	0.2500	0.064*	
C14	0.3390 (5)	0.1916 (5)	0.22919 (12)	0.0541 (11)	
H14A	0.2522	0.2242	0.2428	0.065*	
C15	0.4139 (4)	0.2808 (4)	0.20549 (10)	0.0508 (11)	
H15A	0.3801	0.3730	0.2031	0.061*	
C16	0.5427 (4)	0.2280 (4)	0.18510 (10)	0.0359 (10)	

### Atomic displacement parameters (Å^2^)

**Table:**

	*U*^11^	*U*^22^	*U*^33^	*U*^12^	*U*^13^	*U*^23^
O1	0.0561 (19)	0.085 (2)	0.0526 (18)	−0.0022 (18)	0.0156 (16)	−0.0106 (18)
O2	0.061 (2)	0.068 (2)	0.048 (2)	0.0042 (18)	0.0086 (16)	0.0139 (18)
O3	0.0506 (18)	0.0599 (19)	0.072 (2)	0.0141 (17)	0.0108 (18)	0.0009 (18)
N1	0.0409 (19)	0.0233 (17)	0.052 (2)	0.0028 (16)	0.0007 (18)	0.0074 (17)
N2	0.044 (2)	0.0254 (17)	0.052 (2)	−0.0002 (18)	0.0067 (17)	−0.0009 (19)
C1	0.059 (3)	0.100 (4)	0.081 (3)	−0.011 (3)	0.019 (3)	−0.009 (3)
C2	0.085 (4)	0.074 (3)	0.073 (4)	0.019 (3)	0.007 (3)	0.029 (3)
C3	0.067 (3)	0.096 (4)	0.069 (3)	0.034 (3)	−0.013 (3)	−0.004 (3)
C4	0.034 (2)	0.042 (3)	0.057 (3)	0.004 (2)	0.004 (2)	−0.008 (2)
C5	0.042 (2)	0.031 (2)	0.056 (3)	−0.001 (2)	0.005 (2)	0.002 (2)
C6	0.038 (2)	0.030 (2)	0.042 (2)	−0.002 (2)	0.005 (2)	−0.001 (2)
C7	0.036 (2)	0.032 (2)	0.051 (3)	0.000 (2)	0.002 (2)	−0.003 (2)
C8	0.045 (3)	0.042 (2)	0.040 (2)	−0.003 (2)	−0.001 (2)	−0.003 (2)
C9	0.041 (3)	0.053 (3)	0.040 (2)	−0.006 (2)	0.007 (2)	−0.004 (2)
C10	0.038 (2)	0.027 (2)	0.047 (2)	−0.003 (2)	−0.006 (2)	0.003 (2)
C11	0.033 (2)	0.030 (2)	0.038 (2)	−0.0061 (18)	0.005 (2)	−0.003 (2)
C12	0.047 (3)	0.034 (2)	0.054 (3)	−0.007 (2)	−0.002 (2)	0.006 (2)
C13	0.054 (3)	0.053 (3)	0.053 (3)	−0.010 (2)	0.005 (2)	0.010 (3)
C14	0.050 (3)	0.051 (3)	0.062 (3)	−0.002 (3)	0.017 (2)	−0.005 (3)
C15	0.052 (3)	0.037 (3)	0.064 (3)	0.008 (2)	0.012 (2)	−0.004 (2)
C16	0.040 (2)	0.023 (2)	0.044 (2)	−0.0011 (19)	0.001 (2)	−0.001 (2)

### Geometric parameters (Å, °)

**Table:**

C9—O1	1.379 (4)	C4—C5	1.379 (5)
C1—O1	1.428 (5)	C4—C9	1.403 (5)
C8—O2	1.377 (4)	C5—C6	1.389 (5)
C2—O2	1.422 (4)	C5—H5A	0.9300
C4—O3	1.372 (4)	C6—C7	1.407 (5)
C3—O3	1.427 (4)	C6—C10	1.464 (5)
N1—C11	1.371 (4)	C7—C8	1.383 (5)
N1—C10	1.383 (4)	C7—H7A	0.9300
N1—H1A	0.8600	C8—C9	1.401 (5)
N2—C10	1.329 (4)	C11—C12	1.383 (5)
N2—C16	1.400 (4)	C11—C16	1.419 (5)
C1—H1B	0.9600	C12—C13	1.376 (5)
C1—H1C	0.9600	C12—H12A	0.9300
C1—H1D	0.9600	C13—C14	1.418 (5)
C2—H2B	0.9600	C13—H13A	0.9300
C2—H2C	0.9600	C14—C15	1.376 (5)
C2—H2D	0.9600	C14—H14A	0.9300
C3—H3A	0.9600	C15—C16	1.400 (5)
C3—H3B	0.9600	C15—H15A	0.9300
C3—H3C	0.9600		
			
C9—O1—C1	117.4 (3)	C5—C6—C10	119.9 (4)
C8—O2—C2	118.2 (3)	C7—C6—C10	119.8 (3)
C4—O3—C3	116.9 (3)	C8—C7—C6	119.1 (4)
C11—N1—C10	107.7 (3)	C8—C7—H7A	120.5
C11—N1—H1A	126.1	C6—C7—H7A	120.5
C10—N1—H1A	126.1	O2—C8—C7	124.2 (4)
C10—N2—C16	104.8 (3)	O2—C8—C9	114.9 (4)
O1—C1—H1B	109.5	C7—C8—C9	120.8 (4)
O1—C1—H1C	109.5	O1—C9—C8	118.9 (4)
H1B—C1—H1C	109.5	O1—C9—C4	121.6 (4)
O1—C1—H1D	109.5	C8—C9—C4	119.3 (4)
H1B—C1—H1D	109.5	N2—C10—N1	112.4 (3)
H1C—C1—H1D	109.5	N2—C10—C6	126.4 (3)
O2—C2—H2B	109.5	N1—C10—C6	121.2 (3)
O2—C2—H2C	109.5	N1—C11—C12	132.5 (4)
H2B—C2—H2C	109.5	N1—C11—C16	105.1 (3)
O2—C2—H2D	109.5	C12—C11—C16	122.4 (4)
H2B—C2—H2D	109.5	C13—C12—C11	117.2 (4)
H2C—C2—H2D	109.5	C13—C12—H12A	121.4
O3—C3—H3A	109.5	C11—C12—H12A	121.4
O3—C3—H3B	109.5	C12—C13—C14	121.3 (4)
H3A—C3—H3B	109.5	C12—C13—H13A	119.4
O3—C3—H3C	109.5	C14—C13—H13A	119.4
H3A—C3—H3C	109.5	C15—C14—C13	121.7 (4)
H3B—C3—H3C	109.5	C15—C14—H14A	119.2
O3—C4—C5	123.5 (4)	C13—C14—H14A	119.2
O3—C4—C9	116.4 (4)	C14—C15—C16	117.7 (4)
C5—C4—C9	120.0 (4)	C14—C15—H15A	121.1
C4—C5—C6	120.4 (4)	C16—C15—H15A	121.1
C4—C5—H5A	119.8	N2—C16—C15	130.3 (3)
C6—C5—H5A	119.8	N2—C16—C11	109.9 (3)
C5—C6—C7	120.3 (3)	C15—C16—C11	119.8 (4)

### Hydrogen-bond geometry (Å, °)

**Table:**

*D*—H···*A*	*D*—H	H···*A*	*D*···*A*	*D*—H···*A*
N1—H1A···N2^i^	0.86	2.07	2.918 (4)	169

Symmetry codes: (i) −*x*+3/2, *y*−1/2, *z*.
